# Retraction: Long non-coding RNA FTH1P3 facilitates uveal melanoma cell growth and invasion through miR-224-5p

**DOI:** 10.1371/journal.pone.0282986

**Published:** 2023-03-29

**Authors:** The PLOS ONE Editors

The *PLOS ONE* Editors retract this article [1] because it was identified as one of a series of submissions for which we have concerns about peer review, authorship, and similarities across articles. Image similarities were noted between the GAPDH panel of Fig 4E in [1] and the GAPDH panel of Fig 4J in [2]. These concerns call into question the validity and provenance of the reported results. We regret that the issues were not addressed prior to the article’s publication.

All authors either did not respond directly or could not be reached.
